# To Remove Tonsils

**Published:** 1900-01

**Authors:** 


### To Remove Tonsils.

When removing tonsils, it is often found that there is some difficulty in getting the tonsils within the fenestra of the guillotine. In these cases the tonsil can usually be easily pushed in by pressure on the neck over the site of the tonsil with the fingers of the unoccupied hand.—*International Journal of Surgery.*

